# Integrated miRNA and transcriptome profiling to explore the molecular determinism of convergent adaptation to corn in two lepidopteran pests of agriculture

**DOI:** 10.1186/s12864-021-07905-7

**Published:** 2021-08-09

**Authors:** Sylvie Gimenez, Imène Seninet, Marion Orsucci, Philippe Audiot, Nicolas Nègre, Kiwoong Nam, Réjane Streiff, Emmanuelle d’Alençon

**Affiliations:** 1 DGIMI, Univ Montpellier, INRAE, Montpellier, France; 2 CBGP, INRAE, CIRAD, IRD, Montpellier SupAgro, Univ Montpellier, Montpellier, France; 3 Department of Plant Biology, Uppsala BioCenter and Linnean Centre for Plant Biology, Swedish University of Agricultural Sciences, 75007 Uppsala, Sweden

**Keywords:** Adaptation, Phenotypic plasticity, Genetic convergence, Regulation of gene expression, Insect plant interaction, microRNAs

## Abstract

**Background:**

The degree to which adaptation to same environment is determined by similar molecular mechanisms, is a topic of broad interest in evolutionary biology, as an indicator of evolutionary predictability. We wished to address if adaptation to the same host plant in phytophagous insects involved related gene expression patterns. We compared sRNA-Seq and RNA-Seq data between two pairs of taxa of *Ostrinia* and *Spodoptera frugiperda* sharing maize as host-plant. For the latter, we had previously carried out a reciprocal transplant experiment by feeding of the larvae of the Corn strain (Sf-C) and the Rice strain (Sf-R) on corn versus rice and characterized the mRNA and miRNA responses.

**Results:**

First, we predicted the genes encoding miRNA in *Ostrinia nubilalis* (On) and *O. scapulalis* (Os). Respectively 67 and 65 known miRNA genes, as well as 196 and 190 novel ones were predicted with Os genome using sncRNAs extracted from whole larvae feeding on corn or mugwort. In On, a read counts analysis showed that 37 (55.22%) known miRNAs and 19 (9.84%) novel miRNAs were differentially expressed (DE) on mugwort compared to corn (in Os, 25 known miRs (38.46%) and 8 novel ones (4.34%)). Between species on corn, 8 (12.5%) known miRNAs and 8 (6.83%) novel ones were DE while only one novel miRNA showed expression variation between species on mugwort. Gene target prediction led to the identification of 2953 unique target genes in On and 2719 in Os, among which 11.6% (344) were DE when comparing species on corn. 1.8% (54) of On miR targets showed expression variation upon a change of host-plant.

We found molecular changes matching convergent phenotype, i.e., a set of nine miRNAs that are regulated either according to the host-plant both in *On* and *S*f-C or between them on the same plant, corn. Among DE miR target genes between taxa, 13.7% shared exactly the same annotation between the two pairs of taxa and had function related to insect host-plant interaction.

**Conclusion:**

There is some similarity in underlying genetic mechanisms of convergent evolution of two distant Lepidopteran species having adopted corn in their host range, highlighting possible adaptation genes.

**Supplementary Information:**

The online version contains supplementary material available at 10.1186/s12864-021-07905-7.

## Background

Populations have to keep their ability to survive and reproduce to be maintained upon environmental changes. This is challenging especially when fast changes occur and require adoption of new features, either metabolic, developmental, behavioral, physiological or morphological providing an enhanced fitness, through the process of adaptation. Two main mechanisms of adaptation have been described “phenotypic plasticity” and “adaptive evolution”. Phenotypic plasticity is defined as the ability of organisms to change of phenotype without changing genotype in response to environmental conditions [1]. Since they do not involve mutations, these different phenotypes are expected to involve distinct transcriptional programs. In the case of adaptive evolution, populations can acquire a better fitted phenotype to the environment thanks to the spread in the population of pre-existing genetic variants or of new mutations conferring enhanced capability (For review, [2, 3]).

Phenotypic plasticity represents a fast response to a change in the environment that can occur in many individuals of a population at the same time [4] as opposed to adaptive evolution which may take longer. If the plastic phenotype provides a real gain of fitness in reaction to an environmental change, it may become stabilized by a new mutation and become constitutive, thereby contributing to adaptive evolution. A framework to test this “Plasticity first hypothesis” has recently been proposed and shown to be valid in few biological models [5]. It is better supported in the case study of Amphibians, Spade food toads (*Spea* spp.) with different resource use ecomorphs, in response to alternative diets [6]. The two mechanisms can thus provide complementary responses to natural selection in the environment.

The independent evolution of similar phenotypes between closely related or between distant evolutionary lineages, i.e. -parallelism or convergent evolution, respectively (as defined in [7]) - has been interpreted as highlighting optimized solutions in response to natural selection exerted by similar environments. However, the evolution of similar phenotypes could also reflect constraints exerted by the environment which reduce the range of phenotypic variation. When combined with experimental evidence that the new phenotype provides fitness advantage, the study of parallel or convergent evolution can help unraveling the molecular basis of adaptive traits [8]. Conte et al., who scrutinized examples in the literature, estimated as high the probability that the same genes are involved in parallel or convergent evolution in natural populations [9].

Phytophagous insects are particularly relevant models for the study of convergence and plasticity. Their close specialization to their host plants and the evolution of plants in response to this herbivorous pressure lead to constant adaptive changes in their physiology and behavior.

Some adaptive traits involve few genetic markers like mimicry shifts in Heliconius [10] or specialization to the host in cactophylic *Drosophila pachea* [11]. In other models, comparative genomic approaches uncovered large sets of candidate genes putatively involved in adaptation suggesting a more complex genetic basis ([12] for review). For instance, in the case of adaptation to the host-plants in polyphagous insect pests, comparative genomics or transcriptomics of taxa pairs with different host-plant ranges highlighted large sets of gene families playing a role in different steps of the interaction between the insect and its hosts [13–17]. Among these taxa pairs, we have shown that two of them evolved a fitness benefit compared to proxy of their ancestral lineage when larvae feed on the same host, maize - a major crop plant - in the case of *Spodoptera frugiperda* corn strain and *Ostrinia nubilalis*, the European Corn borer (as compared to *Spodoptera frugiperda* rice strain and *O. scapulalis*, respectively). In this paper, we wish to compare their molecular response to the same host-plant, corn, to know if they adopted similar or divergent paths of phenotypic evolution both at the gene expression or gene function level.

We will focus on the microRNA gene regulator families and on their gene targets. MicroRNAs (miRNAs) are a class of small non-coding RNAs (sncRNAs) of about 22 nt in length, which regulate gene expression at the post-transcriptional level and are known to fine-tune complex genetic networks ([18], for review). miRNAs target mRNAs and interfere with their expression by repression of translation or by acting on mRNA deadenylation and decay resulting in relatively weak expression variation of less than two fold both at RNA and protein levels [18]. Single miRNAs are able to regulate many genes, and single genes can be targeted by several miRNAs resulting in a combinatorial regulation. Regarding miRNA genes evolution, Bartel et al., 2018 estimated that 27 families corresponding to 75 miRNA genes were very broadly conserved since the bilaterian ancestors [19]. In Drosophila, there are 164 miRNAs genes belonging to 104 different families [20]. By comparing miRNA genes content between *Drosophila melanogaster, Blattella germanica, Locusta migratoria, Acyrtosiphon pisum, A. mellifera, Tribolium castaneum, Bombyx mori*, Ylla et al. identified a set of 62 miRNA families common to the insects which they called the “insect microRNA toolkit” [21].

*Spodoptera frugiperda*, also called fall armyworm (FAW), belongs to the superfamily Noctuoidea which includes a large number of agriculture and forest pest species and comprises more than one third of all Lepidoptera. Noctuoidea diverged ca. Ninety-four million years ago (Ma) from the Bombycoidea superfamily [22] which includes the lepidopteran model, *Bombyx mori*. While the latter is monophagous, *S. frugiperda* is polyphagous and a major agricultural pest consisting of two sympatric host-plant strains. The “corn strain” (Sf-C) feeds mostly on maize, cotton and sorghum and the “rice strain” (Sf-R) is mostly associated to rice and various pasture grasses [23]. We have shown by a population genomics analysis that individuals of Sf-C and Sf-R natural populations cluster in two phylogenetic groups [14]. When, in another phylogenetic analysis, a close species was used as outgroup, then the Sf-C individuals grouped and appeared to derive from the Sf-R group [24]. The Sf-R strain can thus be considered as an ancestral proxy-lineage of the Sf-C strain. By reciprocal transplant experiment (RT) on their preferred or alternative host-plants, we showed that the C strain survived clearly better on corn than on rice, survival being a trait directly related to fitness. The Sf-R strain displayed only a non-significant trend to survive better on rice than on corn [13] and seemed more generalist. Using transcriptomic approaches, we characterized molecularly these RT experiments. We showed that some microRNAs and their target genes were involved in phenotypic plasticity or adaptive evolution, this respectively by comparing either the molecular response of each strain on two host plants, or by comparing that of the two strains on the same host-plant [13, 17]. We thus wondered whether the molecular response to the same selective pressure, i.e. the switch to corn as a new host-plant, would involve similar regulatory pathways in other species.

In this paper, we focused on another pair of closely related species of moths [25, 26], the European corn borer, *Ostrinia nubilalis* (On) and the Adzuki bean borer, *Ostrinia scapulalis* (Os), belonging to the genus *Ostrinia* (superfamily Crambidae, Lepidoptera). The first, native to western Europe, Northern Africa, and Western Asia feeds mainly on maize (Gramineae), while the latter, found in Eurasia develops on various dicotyledons including, as major hosts, mugwort (*A. vulgaris L.*), hop (*Humulus lupulus L.*) and hemp (*Cannabis sativa L.*). Based on mitochondrial COII gene (682 bp) sequence phylogenetic analyses [27], On diverged from an ancestral species close to the current Os, and switched to maize following its introduction ~ 500 years ago from America to Western Europe [28]. Larval feeding performance [29] and oviposition preferences [28, 30] suggested that the two species are specialized on these respective host plant sets. In two recent papers, [15, 16], we confirmed these data by reciprocal transplant experiments (RT) showing that at the larval stage, On weighed more, developed faster on corn than on mugwort and showed equivalent survival rate on corn and on mugwort, while Os survived much better on mugwort than on corn. We described the comprehensive repertoire of genes expressed during this larval response to corn and mugwort and sorted the genes according to different classes reflecting plasticity or divergence [15].

**Fig. 1:**
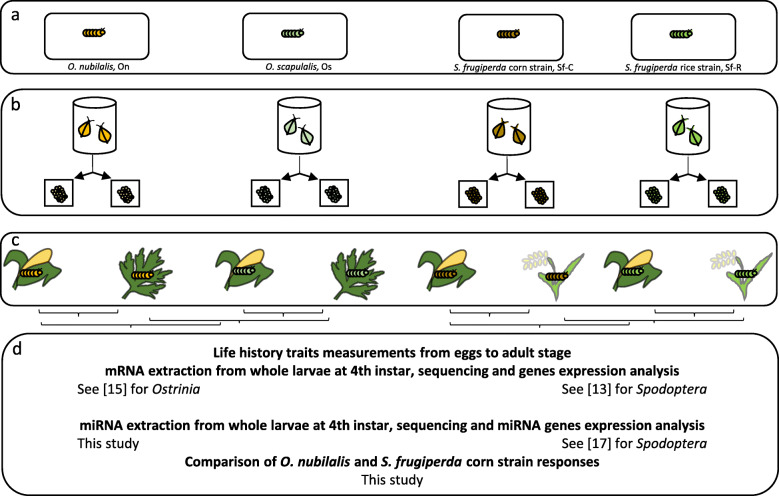
Diagram showing the experimental conditions and the comparisons made. **a** The reciprocal transplant experiments were made with experimental populations reared for one generation on artificial diet after field collection for *Ostrinia* sibling species. They were made using laboratory populations in the case of *Spodoptera frugiperda*. **b** Individual pairs of adults were used for mating and oviposition for the RT experiments. **c** Full-sib eggs have been evenly distributed among replicates on corn or mugwort in the case of *Ostrinia* sibling species, or on corn and rice in the case of *Spodoptera frugiperda*. **d** Larvae were reared from eggs up to the L4 larval stage before mRNA and miRNA extractions. Comparisons made for the differential gene expression analyses are shown by brackets between c and d boxes. Bibliographic references for the complementary datasets used in the present study are shown

To explore further the molecular response to corn of lepidopteran pests, in the same RT experiments involving the two *Ostrinia* sibling species grown on corn or mugwort [15, 16], we isolated and sequenced sncRNAs from feeding larvae. We present the differential expression patterns of miRNAs and of their putative coding genes targets involved i) in phenotypic plasticity of each lineage in response to corn or mugwort or ii) in adaptive evolution or genetic drift, by additional comparison of the two *Ostrinia* species on the same host-plant. Last, we compare the set of microRNAs and of their target genes that are deregulated in the two pairs of taxa of *Ostrinia* or of *Spodoptera* in phenotypic plasticity when feeding on corn or alternative plants or in adaptive evolution or genetic drift, between sibling species on the same plant, the corn. A summary of the experimental design and comparisons made in this study is depicted on Fig. 1.

## Results

### Sequencing and analysis of On and Os small RNA libraries

**Fig. 2:**
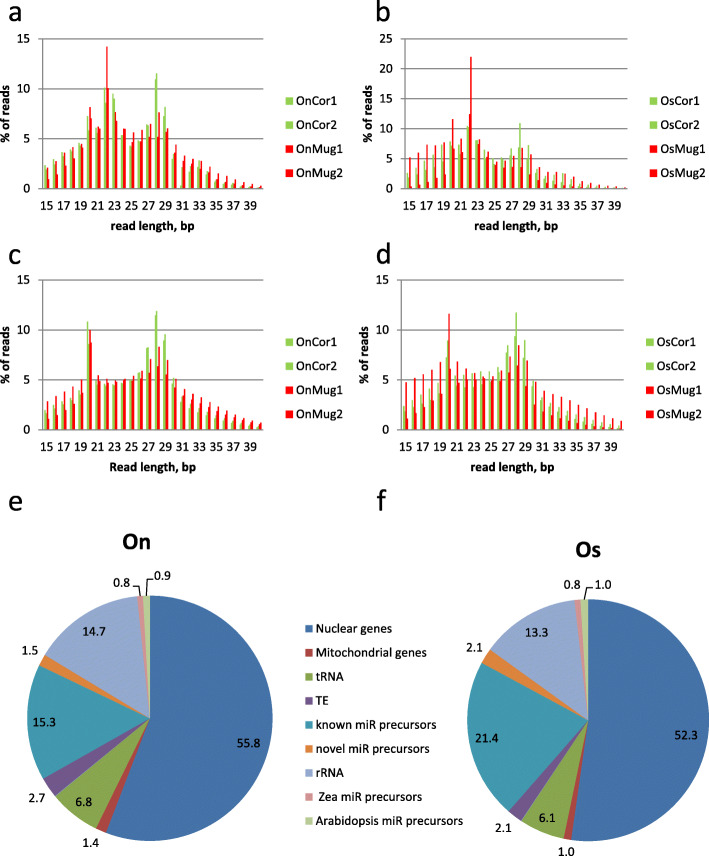
Size profiling of small non coding RNAs and their homology to different RNA classes or to Transposable Elements (TE). The percentage of sncRNA reads is plotted as a function of their size (between 15 nt to 40 nt corresponding to the size range that has been selected from the gel for library construction), panels **a**, **c** *O. nubilalis*, panels **b**, **d** *O. scapulalis*, in green on corn, in red on mugwort. OnCor: On on corn, OnMug: On on mugwort, OsCor: Os on corn, OsMug: Os on mugwort. Panels **a**, **b** total reads, panels **c**, **d** unique reads. Panels **e**, **f** Pie charts representing the average % of reads (total counts from 2 replicates on corn for On (**e**) or Os (**f**)) mapping either to insect or plant miR precursors, or TE (Os TE copies) as expected for putative endo-siRNA or piRNAs, or mRNA, or tRNA, rRNA (18S and 28S RNAs) or mitochondrial genes

To characterize the sncRNAs involved in response to the host-plant or in adaptive evolution of On and Os, we extracted sncRNAs from insect samples collected during a previous reciprocal transplant experiment [16]. A graphic summary of the experimental design can be found on Fig. 1 (left part). The sncRNA extracted from both On and Os larvae reared either on corn or on mugwort (with two biological replicates in each case) were used to construct 8 libraries for Illumina sequencing. Between 10 to 50 Million reads were obtained in each library after adapter trimming and filtering out low quality reads (Additional file 1: Supplementary Table S1) corresponding 0.7 to 2.4 Million unique sequences. Sequences of length in the range 15 to 40 nucleotides were further analyzed. Size profiling obtained with non-collapsed reads (Fig. 2a-b) shows a peak between 21 to 23, corresponding to expression of miRNAs. miRNAs represent on average 24.69+/− 1.30% of the reads on corn, 25.07 +/− 5.56% of the reads on mugwort. Two other peaks of abundance were found in the range of 19–20 nucleotides and 25–33 nucleotides, corresponding probably to other sncRNA classes (siRNAs and piRNAs, respectively). These peaks predominate when size profiling was performed with collapsed reads (unique sequences) (Fig. 2c-b) showing the diversity of pi and siRNAs which may reflect the diversity of Transposable Elements found in On and Os genomes (16.6% of TE in Os genome, [31]) to which most of them are expected to be homolog. MiRNAs are less diverse, representing only 14.37+/− 1.81% of the unique reads on corn, and 15.55+/− 1.15% on mugwort. Sequence homology of sncRNA reads from On or Os to a set of Os reference sequences - nuclear genome, nuclear gene models predicted by Augustus, mitochondrial gene models, tRNAs, rRNAs, TE, known and novel miRNA precursors and to plants miRNA precursors (*A. thaliana* instead of mugwort whose genome sequence is not available and *Zea*) - is shown on Fig. 2d-e for On and Os sRNA reads, respectively. The miRNAs represent 16.8 and 23.5% of the On or Os sncRNA reads that could be annotated by homology to these accessions. We found also less than 1% of reads matching to plants miRNA precursors. This is expected since sncRNA were extracted from the whole body of plant feeding larvae. However since animal and plant miRNAs do not share homology [19] except one family [32], presence of these plant sequences does not interfere with the following study.

### miRNA genes annotation

In [33], a set of miRNA of On have already been described and characterized by homology to known miRNA sequences available in miRBase v.18. However, since the genomic sequences of On (and Os) were not available at that time, the precursors sequences of these miRNAs have not yet been characterized. In our paper, we used miRDeep2 software to detect miRNA genes from the Os genome assembly OSCA v1.2 [31] (See Material & Methods). As shown on Table 1, we identified 67 (from On reads) and 65 (from Os reads) known miRNA genes among which 33 were new compared to those already published [33]. Most of novel miRNA genes identified in our study (196 from On reads, 190 from Os reads – the term “novel” referring to miRNA mature sequence which were not recorded in miRbase) were not published in [33]. The list and sequences of miRNA gene precursors and mature miRNAs are available on Supplementary Excel Table S2 (see Additional file 2) as well as the correspondence between On or Os reads based predictions. The study of nucleotide composition showed that all mature miRNAs predicted using On reads are enriched in uridine (U) at position 1 as well as mature known miRNAs predicted from Os reads (Additional file 3: Supplementary Figure 1, top panels). This is an expected feature of mature miRNA sequences [34]. However novel miRNA predicted from Os reads present equivalent C or U content at position 1 (Additional file 3: Supplementary Figure 1, bottom panel).

**Table 1:** miR genes number in On and Os

Species	Known	New compared to Yu^a^	Known and unique	Known Score > 4 (+randfold yes)	Novel	New compared to Yu et al.^a^	Novel And unique	Novel Score > 4 (+randfold yes)
On	67	33	53	56 (55)	196	190	164	106 (105)
Os	65	ND	51	52 (51)	190	ND	161	95 (91)

^a^Since *the Ostrinia* microRNAs predicted in [33] are not registered in miRBase, we checked whether each of our predicted miRs had already been predicted

### Differential miRNA expression between mugwort and corn or between on and Os on the same plant

Since miRNAs play important roles in many biological processes, we supposed that the expression of miRNAs might be regulated upon a change of diet in *Ostrinia* sibling species larvae.

#### Between mugwort and corn

**Fig. 3:**
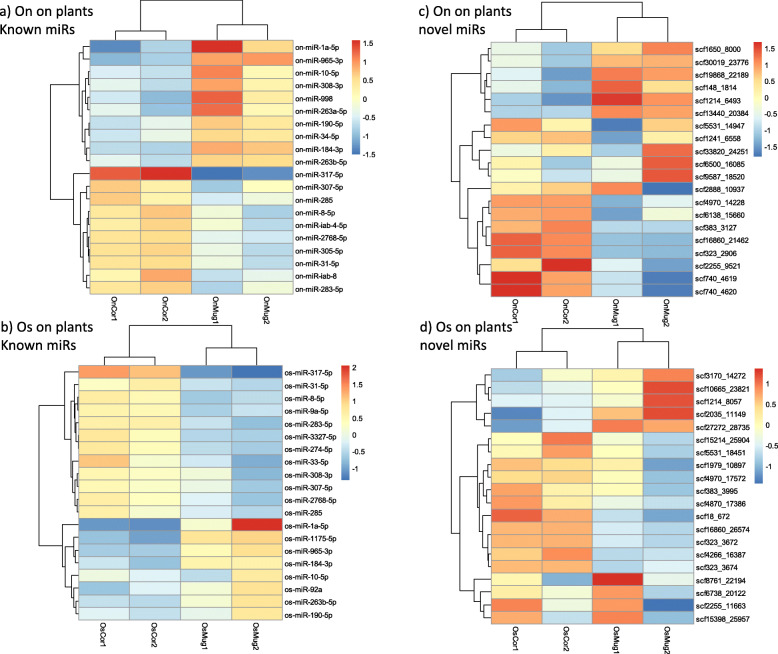
Heatmaps showing differential expression of miRNAs genes on mugwort compared to corn in *Ostrinia* larvae (L4 instar), after rearing on whole plants. The 20 miRNAs showing the most significant differential expression after DESeq2 analysis (log2 fold change > 1 or < 1 and FDR < 0.05) are shown. OnCor: On on corn, OnMug: On on mugwort, OsCor: Os on corn, OsMug: Os on mugwort. Figure 3a) On on plants, known miRs Fig. 3b) Os on plants, known miRs. Figure 3c) On on plants, novel miRs Fig. 3d) Os on plants, novel miRs

The global expression of miRNAs after feeding on corn or mugwort was profiled using DESeq2 [35], a software that enables differential expression study of miRNAs between different conditions based on counts of sequences mapping to precursors of miRNAs defined previously. On Supplementary Figure 2 (Additional file 4), the log2 fold change for each miRNA gene is shown as a function of mean expression i.e. the average of counts normalized by size factors. Red dots correspond to miRNA genes that are significantly differentially expressed (FDR less than 0.05) on mugwort compared to corn. We also visualized samples (treatments, replicates) by Principal Component Analysis (PCA; Supplementary Fig. 3, Additional file 5) for known and novel miRNAs of On (top) and Os (bottom). In each case, we found more variation between treatments, i.e.*,* change of host-plant, than between biological duplicates. Based on read counts 37 out of 67 known miRNAs (55.22%) and 19 out of 193 novel miRNAs (9.84%) were DE in On reared on mugwort compared to On reared on corn (19 out of 37 known miRs and 7 novel ones were upregulated, the rest being downregulated; DE miR genes are shown as red dots on MA plots on Supplementary Figure 2, Additional file 4) detailed results of DESeq2 analysis can be found in Supplementary Excel Table 3, Additional file 6). Twenty five out of 65 known miRNAs (38.46%) and 8 out of 184 (4.34%) novel miRNAs were DE in Os on mugwort compared to corn (11 out of 25 known miRs and 3 novel ones were upregulated; detailed results of DESeq2 analysis can be found in Supplementary Excel Table 3, Additional file 6). Heatmaps representing the expression level of the most DE known and novel miRNA genes are shown on Fig. 3a, c On, b, c Os. Using TaqMan RT-qPCR miRNA assay, we could validate experimentally the upregulation of miR-1a, miR-34, miR-199 on mugwort compared to corn in On and of miR-1a in Os on the same plant (Table 2). However, for miR-317, for an unknown reason, we failed to validate its upregulation on corn by RT-QPCR both in On and Os. The presence of an RNA molecule may compete with the miRNA for the TaqMan probe in these conditions.

**Table 2:** Validation by real time PCR of variation in microRNA expression

Experimental condition	MicroRNA	Relative expression	StdError	95% C.I.	P(H1)^a^	Result
On (Mugwort/Corn)	miR-124 (Ref)	1				
	miR-1	7.495	(4.141–15.144)	3.252–21.391	< 0.001	UP
	miR-34	2.231	(1.500–3.306)	1.227–4.060	< 0.001	UP
	miR-190	4.428	(3.777_5.169)	3.418–6.828	< 0.001	UP
	miR-317	1.799	(1.158–2.810)	0.929–3.626	< 0.001	UP
Os (Mugwort/Corn)	miR-124(Ref)	1				
	miR-1	2.189	0.895–5.399	0.712–6.493	0.011	UP
	miR317	1.146	(0.877–1.529)	0.745–1.870	0.102	

The relative expression of miR genes depending on the host-plant (on mugwort compared to corn) in either the On or the Os sister species

^a^P(H1) - Probability of alternate hypothesis that difference between sample and control groups is due only to chance

#### Between On and Os on corn

**Fig. 4:**
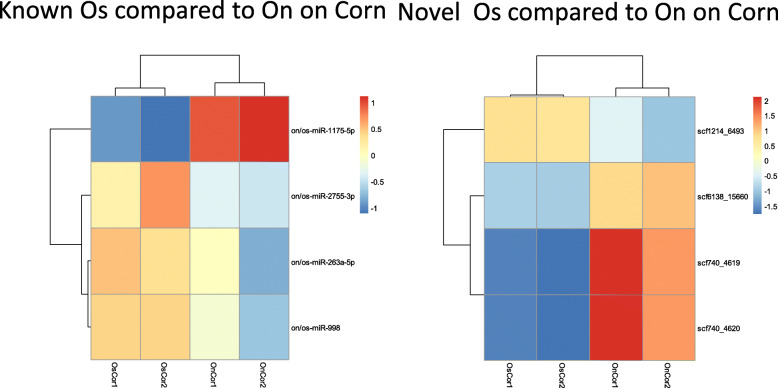
Differential expression of miRNAs genes according to the genetic background. Heatmaps showing the relative expression of miRNAs in Os compared to On on corn is shown, for known miRNAs (left) or novel ones (right). OnCor: On on corn, OnMug: On on mugwort, OsCor: Os on corn, OsMug: Os on mugwort

Genetic drift or selection by the environment may have led to constitutive changes in expression of miRNAs and their target genes between the two strains. We analyzed by DESeq2 the variation in miRNA expression between On and Os on the same plant. On Supplementary Figure 4 (Additional file 7), from the study of log2 fold change we can observe that both some of the known and of the novel miRNAs are DE between Os and On on corn. More precisely from the detailed DESeq2 results presented in Supplementary Excel Table S4 (Additional file 8), we can see that 8 out of 64 (12.5%) known miRNAs are DE between On and Os on corn and 8 out of 117 (6.83%) novel miRNAs are DE between sibling species on corn. The analysis of variation between samples by PCA shows that there is more variation between conditions, i.e., genetic background, than between biological duplicates (Supplementary Figure 5, Additional file 9). The expression level of the most differentially expressed miRNAs is shown on Fig. 4.

On mugwort, we did not find significant variation in miRNA expression between the two *Ostrinia* species except for one of the novel miRNAs, scf13357_20354, with a fold change of -1.90 at an FDR of 0.025 in Os compared to On (Supplementary Excel Table S4 (Additional file 8).

### Expression differences both between sibling species and between plants

**Fig. 5:**
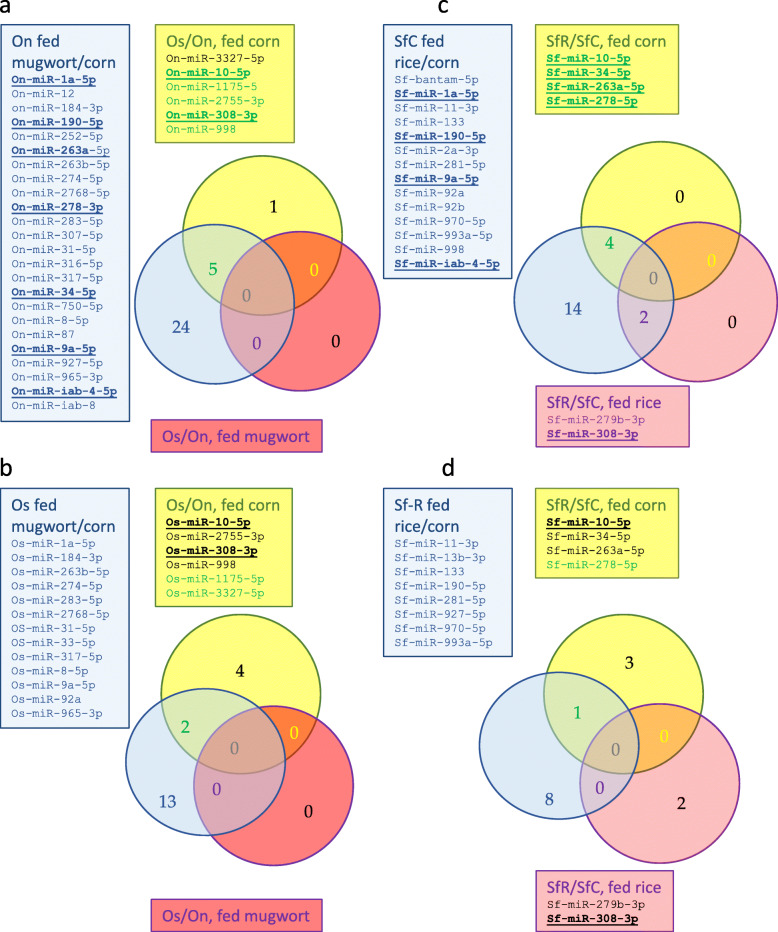
Are the constitutive expression differences between sibling species involved in phenotypic plasticity within On or Os? This Venn diagram highlights the miRNAs (known class) that are differentially expressed (FDR < 0.05) both between sibling species on the same plant and within *Ostrinia* species (green, purple or orange characters, On: panel **a**, Os: panel **b**) on mugwort compared to corn. Comparison of this response with that of the two strains of *Spodoptera frugiperda*, the corn strain Sf-C and the rice strain Sf-R. The Venn diagram highlights the miRNAs that are differentially expressed (FDR < 0.05) both between strains on the same plant and within strain (green, purple or orange characters, Sf-C: panel **c**, Sf-R: panel **d** on rice compared to corn. Underlined: the miRNA whose differential expression is shared by the two pairs of taxa

The miRNA expression differences between the two Ostrinia species may result from genetic variation occurring by drift or following adaptive evolution following an environmental change, in this case possibly the different host-plants. To identify miRNA genes of the known class putatively involved in adaptive evolution in response to the host-plant, we looked for those showing constitutive expression differences between On and Os on the same plant as well as a variation in their expression in response to a change of host-plant (FDR < 0.05). As shown on Fig. 5a, 5 out of 6 known miRNAs showing constitutive variation between sibling species on corn are also regulated in On upon a change of plant suggesting a role for miRNAs in adaptive evolution of On. These are miR-10-5p, miR-1175-5p, miR-2755-3p, miR-308-3p, miR-998. Only two out of the 6 miRNAs constitutively regulated between sibling species show also variation in Os according to the diet, miR-1175-5p and miR-3327-5p (Fig. 5b).

### Comparison of the miRNA responses in two lepidopteran pests of corn, On and Sf-C

Evolutionary convergence can guide the identification of molecular players involved in adaptation to the same environmental change. Since both On and Sf-C during their evolution history adopted the same host-plant, corn, we started to compare their molecular response first at the level of miRNA, then at the level of the targets of these miRNAs. A summary of the experimental design and dataset used for this study is presented on Fig. 1.

On the Venn diagrams shown on Fig. 5, nine miRNAs that are regulated according to the host-plant or the genetic background both in *Ostrinia nubilalis* and *Spodoptera frugiperda* Corn strain (Fig. 5a, c) are underlined, these are miR-1a-5p, miR-10-5p, miR-190-5p, miR-263a-5p, miR-278-3p, miR-34-5p, miR308-3p, miR-9a-5p, miR-iab-4-5p. Only two miRNA among those regulated according to the host-plant or the genetic background are shared between *Ostrinia scapulalis* and *Spodoptera frugiperda* Rice strain (miR-10-5p and miR-308-3p underlined in Fig. 5b, d).

### Identification of miRNA gene targets

A comprehensive analysis of the gene targets of all known miRNA described in this study was performed using two software - TargetScan and miRanda - on On and Os RNA contigs containing a 3’UTR (See Material & Methods). The overlapping outputs by the two software are available on Supplementary Excel Table 5, Additional Table 10. We found 2953 On and 2719 Os unique genes that are targeted by one or more miRNAs, 3020 for Sf-C.

### Common miRNA targets genes involved in two lepidopteran pests of corn On and Sf-C

We wondered if the adoption of the same host-plant, corn, had led to the evolution of similar molecular responses in different insect pairs of species sharing corn as host-plant (i.e.*,* Os versus On*)* and Sf-R versus Sf-C. In order to compare the expression data with the same method, we re-analyzed the gene expression data published previously in the same RT experiment in [15] for On and Os using DESeq2 instead of EdgeR. For *Spodoptera frugiperda*, the expression data of coding genes and their analysis by DESeq2 has been published in [13]. Known miRNAs involved in the RT experiment have been described [17] and in this first paper, Moné et al. focused on the gene targets of the most DE miRNAs only. A summary of the comparisons and datasets used is presented on Fig. 1. We provide now a comprehensive analysis of the targets of all the known Sf miRNA identified by TargetScan and miRanda as done for *Ostrinia* (Additional file 10: Supplementary Excel Table 5).

Among the gene models showing significant variation in their expression in the same RT experiment as the miRNAs, we identified those predicted as miRNAs targets. We provide lists of DE target genes (FDR < 0.05) of known miRs predicted by TargetScan which were also predicted as known miRNAs targets by miRanda (Additional files 11 & 12: supplementary Excel Tables 6 & 7, for On on plants and Os vs On on corn, respectively, Additional file 13: supplementary Excel Table S8 for *S. frugiperda*). This represented DE genes that are predicted as target of the same miRNA by both software many of which are targeted by multiple miRNAs.

Respectively 54 and 263 different miRNA target genes showing variation in their expression according to the host-plant have been identified in On and Sf-C (In *Ostrinia*, we compared target gene expression on mugwort compared to corn, in *Spodoptera*, we compared target gene expression on rice compared to corn, the expression variation corresponds to Log2 fold change in Supplementary Tables 6, 7). Most of them are targeted by multiple miRNAs. We compared their gene annotation in On and Sf-C. Twenty-four On miRNA targets showed similar functional annotation compared to Sf-C ones, and they could be sorted in seven functional classes (i) digestion, metabolism, feeding behavior (ii) detoxification (iii) immunity (iv) chemosensory genes (v) development (vi) transport (vii) other. Eight shared exactly the same terms in their annotation as Sf-C miR targets, a PGRP and a serine protease inhibitor, a fatty-acyl CoA reductase, a cuticular protein (RR-2 motif 127), three transporters, alpha tocopherol transfer protein, organic cation transporter protein and facilitated trehalose transporter Tret1, as well as a laccase.

Respectively 344 and 796 different miRNA target genes showing variation in their expression according to the genetic background have been identified in On and Sf-C (We compared the target gene expression in Os relative to On when fed on the same plant, and in *Spodoptera,* we compared it in Sf-R relative to Sf-C). One hundred eleven On miRNA gene targets showed similar annotation with Sf-C ones, and 47 shared exactly the same annotation with 8 belonging to class 1(a takeout protein, two lipases, three enzymes involved in fatty acid or sugar metabolism), 4 to class 3 (a PGRP, a Toll-like receptor, a mucin, an hemolymph protein), 2 to class 4 (antennal esterase and FAR), 8 to class 5 (among which chitinases, ecdysone oxidase), 10 to class 6 (among which alpha tocopherol transfer protein, organic cation transporter protein and facilitated trehalose transporter Tret1), among the rest, protein yellow. For a summary, the DE miR targets shared between *Ostrinia* and *Spodoptera* are listed in supplementary Excel Table 9 (additional file 14).

## Discussion

In this paper, we characterized the set of miRNAs and their target genes that are involved in the plastic response of the two *Ostrinia* species following a change in their environment, i.e., when fed on their preferred or alternative host-plants, mugwort versus corn. We also identified the miRNA genes and their targets that are constitutively deregulated between the two sibling species on the same host-plant, whose differential expression likely results from adaptive evolution or genetic drift. Finally, to highlight genes involved in convergent evolution, we qualitatively compared the molecular response to host-plant change of On with that of another corn pest Sf-C, that we had characterized previously phenotypically and molecularly [13, 17]. We also compared the constitutive differences at the level of miRNAs and their target genes between the two pairs of taxa Os versus On and Sf-R versus Sf-C. We found molecular evidences supporting convergent evolution in response to host-plants.

### Comparison with Yu & Coates paper

We predicted 67(65) known miRNA genes in On (and Os) and 196 (190) novel ones. Yu et al. compared the relative expression of miRNAs and their target genes between two *O. nubilalis* strains, resistant versus susceptible to Bt toxin [33]. Compared to Yu el al., we found in On 33 additional known and 190 additional novel miRNAs sequence (we extracted the sncRNAs from the whole larvae of On, while they analyzed gut specific ones). We provide the accurate precursor sequence thanks to the availability of Os reference genome sequence [31], while they based their analysis on precursor sequences from *Bombyx* in miRbase.

Insects often use similar strategies to detoxify plant specialized metabolites or insecticides [36], therefore the miRNAs identified in our study can also be implicated in pesticide resistance. Yu et al. found miR-31 and mir-9b-3p as the most upregulated miRs in Bt resistant versus susceptible strain and miR-263b-5p and miR-306 the most downregulated ones: Interestingly miR-31 and mir-263b-5p are also among the most deregulated miRs in On and Os upon a change of host-plant (Fig. 2) and miR-263b-5p is also deregulated between On and Os on corn (Fig. 3). Moreover, among miR263b-5p DE gene targets, we found an homolog of CYP6T1 from *Chilo suppressalis* which led us to conclude that these miRNAs may indeed function in regulating detoxification target genes. We comment further on this gene later in this section.

### Variation of miRNA expression according to plants or between sibling species on the same plants

Our read counts based analysis showed that 37–55.22% - On known miRNAs (25–38.46% - in Os) and 19–9.84% - novel On miRNAs (8–4.34% - Os) were DE on mugwort compared to corn. To highlight constitutive expression difference, the comparison of read counts between *Ostrinia* species on the same host plant showed that 8–12.5%- known miRNAs and 8–6.83%- novel ones are DE between *Ostrinia* species on corn, while only one novel miRNA showed significant expression variation between them on mugwort. The miRNAs expression differences that we uncovered between the sibling species may result from genetic drift and also possibly from divergent selection by the environment, in this case the different host-plants. To identify the latter, we searched for the miRNAs that were differentially expressed both between On and Os on the same plant and within lineages in response to the host-plant. Five out of six known miRNAs showing constitutive variation between *Ostrinia* species on corn are also regulated in On upon a change of plant suggesting a role for miRNAs in adaptive evolution of On. These are miR-10-5p, miR-1175-5p, miR-2755-3p, miR-308-3p, miR-998. Only two out of the six miRNAs constitutively regulated between *Ostrinia* species show also variation in Os according to the plant, miR-1175-5p and miR-3327-5p (Fig. 5). Among them, microRNA-998-3p contributes to Cry1Ac-resistance by targeting ABCC2 in three lepidopteran insects [37].

Our experimental design was based on two replications in each condition. This number of replication is a minimum for miRNA-Seq analysis according to the ENCODE current standards. Using the Euclidean distance between samples, we could show that our samples cluster first by species then by experimental conditions (not shown). However, this number of replicates is not sufficient to determine precisely the biological variability in our RT experiment and thus to optimize the power and sensitivity of our statistical analysis of miRNA expression. Lamarre et al. showed that the rate of false positives obtained with DESeq2 is minimal with 2 replicates and increases with the number or replicates [38]. They recommend the threshold of 0.25 for two replicates (2^-r^, with r the number of replicates) to enhance the sensitivity and specificity of DE analysis. By having chosen the standard FDR threshold of 0.05, we may have missed information about miRNA expression. According to them, 70% of true positives can be detected with two replicates and this number increases with the number of replicates. We conclude that our experimental design enabled detection of a reasonable number of reliable DE miR candidates although more repetitions may be necessary to deepen the study.

### Comparison between on and sf at the miRNA level

The number of unique miRNA genes from *O. nubilalis* annotated during this study is close to that described in Sf-C genome, 53 and 57, respectively (known class), 196 and 139 (novel ones). Our annotation may not reflect the whole repertoire of miRNA genes in each species. The number of miRNAs per insect species ranges between 100 and 200, although *B. mori* is an exception with 487 reported miRNAs [21]. Among the miRNA genes of known class annotated in our study, 45 were shared between On and Sf-C, which represent 85 and 79%, respectively, Supplementary ExcelTable 10, Additional file 15. We can conclude that larval development on host-plants in the two pest insects involves a very similar repertoire of miRNAs. This high similarity suggests existence of a strong conservation of the miRNA repertoire in insects or of some level of genetic convergence between the two species in their response to similar environments, since this annotation relies on mapping of sncRNA sequences originating exclusively from larvae reared on plants, both in On and in Sf-C and not from other experimental conditions. A set of miRNAs conserved between distant insect species has been identified as the “insect miRNA toolkit” [21], it comprises 62 known miRNAs among which 34 (54.8%) were annotated in On in this study (in Sf-C 39, (62.9%)). We then wondered if among annotated miRNA genes in this study, some were shared between Sf-C and On but were not listed in this insect miRNA toolkit, and could be more specifically expressed in these two insect corn pests, and thereby more likely to reflect genetic convergence between them. Among them, we found miR-263-a-5p, miR-263-b-5p, miR-274-5p, miR-285, miR-307-5p, miR-308-3p, miR-3338-5p, miR-932, miR-993a-5p, miR-998.

We then focused on the miRNAs shared between *Ostrinia* and *Spodoptera* and showing differential expression either upon a change of host-plant or depending of the genetic background (Os versus On or SfR versus SfC).

We found that nine out of thirty miRNAs are regulated according to the host-plant or to the genetic background both in *Ostrinia nubilalis* and *Spodoptera frugiperda* Corn strain (underlined on Fig. 5 a, c), these are miR-1a-5p, miR-10-5p, miR-190-5p, miR-263a-5p, miR-278-3p, miR-34-5p, miR308-3p, miR-9a-5p, miR-iab-4-5p. Only two out of 19 regulated miRs, miR-10-5p and miR-308-3p, are shared between Os and Sf-R (Fig. 5b, d). This suggested possible convergence in regulation evolution between the taxa sharing corn in their host-range. Since the functional study of miRNA in Lepidoptera is still in its infancy and only few pieces of knowledge are available on their biological role, we started to analyze their gene targets, whose annotation was expected to be more informative of the biological regulatory pathway involved.

### Comparison at the miRNA targets level

We first comment on all miRNA DE gene targets which were shared between Sf-C and On. These targets can be regulated by different or identical miRs in *Ostrinia* compared to *Spodoptera*.

We then comment on those among them which are specifically targeted by the same miRNAs showing differential expression in our RT experiments according to the host plant or to the genetic background in the two pairs of lineages (Os versus On and Sf-R versus Sf-C) which are underlined on Fig. 5.

### Common targets involved in plastic response to a change of host-plant

Interestingly, facilitated trehalose transporter Tret1, the target of two most DE miRNAs in Sf-C on corn compared to rice is also a DE miRNA target in the response of On to host-plants. In most insects, Trehalose (a-D-glucopyranosyl-(1,1)-a-D-glucopyranoside) is the main sugar in the hemolymph of most insects. The transport of trehalose produced in Drosophila fat body and its uptake into other tissues is performed by Tret 1, allowing regulation of trehalose levels in the hemolymph [39]. Proteins containing a CRAL_TRIO domain, among which alpha-tocopherol transfer protein, bind small lipophilic molecules such as retinal, inositol. In Lepidoptera, this gene family has expanded and may be involved in the evolution of visual systems [40]. Laccase (EC 1.10.3.2) belongs to a group of multicopper oxidases specific for polyphenols and aromatic amines. This enzyme is involved in cuticle sclerotization leading to hardening of the insect exoskeleton [41]. Like cuticular proteins, they are required for the developmental process [42]. Fatty acyl reductases (FARs) are involved in the biosynthesis of fatty alcohols which play various biological roles. Insects typically harbor numerous FAR gene family members. Some FARs are known to be involved in pheromone biosynthesis, however the biological role of a large number of FARs in insect genomes is still unknown [43]. Several serine protease inhibitors are contained in insect hemolymph, like in vertebrate serum. Serine protease inhibitors can be involved in insect anti-microbial defense mechanisms, development, metamorphosis and digestion [44]. Essential roles in the immune systems of insects and higher animals are played by Peptidoglycan recognition proteins (PGRPs), protect them against pathogens including bacteria [45].

Among these miRNAs targets, a subset is targeted by the same DE miRNAs in Sf-C and On upon a change of host-plant (Fig. 5) (miR-1a-5p, miR-10-5p, miR-190-5p, miR-263a-5p, miR-278-5p, miR-34-5p, miR-308-3p, miR-308-3p, miR-9a-5p, miR-iab-4-5p). Among them, were found the three transporters Tret1, Alpha tocopherol transfer protein, organic cation transporter as well as a Laccase, a glycine rich cuticular protein and two FARs. We have also two On P450 genes, homolog of CYP4M39 and CYP6CT1 from *Chilo suppressalis*. In Sf-C, their best homologs by BlastX against OGS2.2 have been annotated as CYP4M15V2 and CYP6CT1 (annotator F. Hilliou, GSSPFG00018886001.3-PA gene = CYP4M15V2 and GSSPFG00018442001.3-PA gene = CYP6CT1 [14]). CYP6T1 belongs to clan 3 of P450 involved in detoxification of phytochemicals [46]. CYP4M15 belongs to clan 4. CYP4 family members are involved in odorant metabolism [47] as well as in cuticular hydrocarbon biosynthesis [48].

### Common targets involved in adaptive evolution or genetic drift

Among the miRNA targets that are DE in response to the host plants both in On and Sf-C and that we described in the previous section, six are also deregulated constitutively between the two pairs of taxa: a PGRP, a fatty-acyl CoA reductase, three transporters, alpha tocopherol transfer protein, organic cation transporter protein and facilitated trehalose transporter Tret1, as well as a laccase. This suggests that these genes are important candidates involved both in adaptive phenotypic plasticity and adaptive evolution.

Among the other DE miRNA targets shared between *Ostrinia* and *Spodoptera*, UGTs could be interesting as gene candidates involved in adaptive evolution because they are acting on toxic molecules specifically associated to corn. Some grasses (Poaceae) produce benzoxazinoids (BXDs) to deter herbivores, these are wheat, rye, and maize, while others do not, like rice, oat, sorghum, and cultivated barley ([49] for review). Upon attack of plant tissue by a chewing herbivore for instance, specific plant β-glucosidases can hydrolyze these indole-derived compounds and release toxic aglucones [50]. Woulters et al., 2014 described that *Spodoptera* species reglucosylate the aglucone DIMBOA derived from the most abundant BXD in maize leaves, (2R)-DIMBOA-Glc, as a detoxification strategy [51]. Thanks to the stereoselectivity of this conjugation, the new glucoside, (2S)-DIMBOA-Glc, become resistant to plant glucosidases, which can only hydrolyze the plant derived(2R)-DIMBOA-Glc. Both insect- and plant-derived UGTs glucosylate BXDs using UDP-Glc but, with a different final stereochemistry. Several Lepidopteran species such as *S. frugiperda*, *S. littoralis*, *Mythimna separata* and *Ostrinia furnacalis* have been previously proposed to perform this glucosylation of BXDs [52–55]. Another detoxification mechanism has been described in *S. frugiperda* and *S. littoralis* [56] based on N-glucosylation of MBOA, a toxic spontaneous degradation product of other BXDs. In his thesis, [57], Woulters identified 39 putative UGTs from *S. frugiperda*, successfully expressed 25 of these in *Trichoplusia ni* cells, and screened them for BXD-UGT activity. He showed that DIMBOA-UGT sequences are present in family UGT33, and MBOA-UGT sequences, in families UGT40, UGT42, and UGT46. The UGTs that we identified in this study that are miR targets in On or Sf-C belong to UGT families 33, 40, 46: In Sf-C, miR-13a-5p targets GSSPFG00031881001 (Name UGT33–01 symbol UGT33–04, annotated as close to HaUGT33J1 by M Maibeche and SJ Ahn [14] itself homolog to UDP-glycosyltransferase 33 J2 of *S. littoralis*). In *H. armigera*, UGT33J1 is expressed specifically in the cuticle of the larval body [58]. It may glycosylate endogeneous cuticle tanning precursors [59]. In On, On-miR-14 (scf3239_14373) targets isotig18003 whose best homolog at nr is HaUGT40M1, expressed in midgut and fat body in *H.armigera* [58]*.* In Sf-C, GSSPFG00021909001 is targeted by Sf-miR-252-5p (superscaffold_630_32959) which is annotated as close to HaUGT46A4 and also homolog to UDP-glycosyltransferase 46A6 of *S. littoralis* (close to HaUGT46A3). In *H. armigera*, UGT46A4 and UGT46A3 differ only in sequence of exon 1 and likely result from alternative splicing of the same gene [58]. In *S. littoralis*, the restricted expression of UGT46A6 in the antennae suggests that this gene might be specifically involved in chemoperception or in maintaining chemosensory organ homeostasis. However UGT46A6 expression is regulated by Z3–6:Ac, a green leaf volatile used by both insect sexes as a chemical cue to locate the host plant. It is also regulated by the insecticide deltamethrin but in opposite way compared to Z3–6:Ac [60]. This suggests that it is involved in detoxification of airborne toxic volatiles since it is expressed in antennae. To our knowledge however, the activity of these UGT enzymes against BXD has not been tested.

Among the other targets which are shared between *Ostrinia* and *Spodoptera*, two were annotated as takeout and yellow protein. Take-out, a representative of the takeout gene family, is putatively involved in feeding behavior and response to starvation as in *D. melanogaster* [61]. The Drosophila *yellow* gene is related to normal larval and adult pigmentation and movement, and the mating behavior of male and female [62], however the *Drosophila melanogaster yellow* gene family consists of a total of more than 14 genes whose function are not known. The seven members of the *Bombyx* yellow protein family have a high transcription level in ovary and testis. This suggests that Bm yellow protein family were also involved in reproduction [63]. Genes involved in development of the insect like ecdysone oxidase and chitinases were also shared. Insect chitinases belong to different groups and serve non redundant functions, they are essential for insect survival, molting or development [64].

When we focus on the On genes targeted specifically by miR-10-5p and miR-308-3p, the two miRNA that are constitutively DE between the two pairs of lineages (Fig. 5), we find in On, a FAR, rost, a serine protease, a CRAL_TRIO domain protein corresponding putatively to the alpha-tocopherol transfer protein. Three genes are also identified as targets of these miRNAs in Sf-C, these are the FAR gene, a gene encoding an innexin and a scavenger receptor of class B. Innexins are necessary for intercellular communication and play important roles in invertebrates mainly in development [65].

A large range of developmental and physiological processes are regulated by steroid hormones in higher organisms. These hormones are synthesized from cholesterol in the adrenal gland, ovaries or testes in mammals. In these tissues and in the liver, the Scavenger Receptor Class B type I (SR-BI) is one of the receptors playing a role in the selective uptake of cholesterol, mainly in the form of High Density Lipoprotein cholesteryl ester (HDL-CE). SR-BI as well as CD36, CLA1 and LIMPII belong to a family of proteins with two- transmembrane domains called the “Cluster of Differentiation 36” (CD36) family, which are often referred to as fatty acid transporters or Scavenger Receptors. In *D. melanogaster*, the majority of the fourteen CD36 genes identified are uncharacterized. Some of them, such as croquemort (crq), epithelial membrane protein (emp), neither inactivation nor afterpotential D (ninaD), peste (pes), scavenger receptor acting in neural tissue and majority of rhodopsin is absent (santa-maria) are related to a variety of functions, such as autophagic cell death, the immune response, cell adhesion, phototransduction [66].

### Molecular convergence matching phenotypic convergence

Our miRNA and transcriptome profiling highlighted gene expression patterns matching phenotypic convergence i.e., adoption of the same host, corn, in two pairs of Lepidopteran taxa. Since the 3’UTR is not known for all genes neither in *Ostrinia* nor in *Spodoptera*, our analysis is not exhaustive, however we can comment on the proportion of DE genes targets with shared annotation between pairs of taxa. While we could uncover about 3000 unique gene targets of the known miRs identified in that study, 344 were DE between sibling species on corn, among which 111 (32.3%) were DE and shared similar annotation between *Ostrinia* and *Spodoptera* on corn (13.9% if we take Sf-C as reference), 13.7% of the DE genes shared exactly the same annotation between the two pairs of taxa (5.9% if we take Sf-C as reference). They could be sorted in six functional classes compatible with playing a role in interaction with the host-plant: (i) digestion, metabolism, feeding behavior (ii) detoxification (iii) immunity (iv) chemosensory genes (v) development (vi) transport. Similarly, a comparative transcriptomic study has been performed between two pairs of taxa of spiders in the Canaries island sharing in each case a generalist and a specialist [67]. The two specialists’ species of their study showed modifications in their mouthparts that have been associated with a preference for using isopods as a prey despite of its toxicity. They could identify a set of hundred genes sharing expression patterns between the two pairs of taxa whose gene functions was in accordance with the ability to detoxify the preys, putative molecular substrates of convergent evolutionary changes [67]. Their hypothesis is supported by presence of signatures of positive selection in some of the pairs of orthologs sharing expression patterns. A population transcriptomic study has been performed in nine-spined sticklebacks in pair of species reflecting marine versus fresh water habitats and compared with gene expression data from pairs of taxa of three-spined sticklebacks from similar environments [65]. Depending on the tissue studied between 1000 to 1500 genes were DE in nine-spined sticklebacks according to a change of environment among which 5% were also DE in three-spined sticklebacks. The idea that similar molecular solution can occur repeatedly during adaptation to similar environment is supported by experimental evolution [68, 69]. We believe that identification of these molecular actors in pests of maize and of their regulators can help our understanding of how an insect becomes a pest of agriculture as well as the design of new strategies to control these populations.

## Material & Methods

The present paper is based on a combination of i) published datasets (*Spodoptera* RT [13] and miRNA [17]) ii) published datasets reanalyzed here (*Ostrinia* RT [15]), iii) a new dataset of miRNA obtained for *Ostrinia* samples and detailed hereafter (See Fig. 1).

### Reciprocal transplant experiment and biological material

The molecular analysis performed in this paper is based on moth samples collected in a previous reciprocal transplant experiment in which we had described phenotypic traits of On and Os on their preferred and alternative plant [16]. Briefly, fertile egg masses of the two moth species On and Os were used to infest maize and mugwort plants in outdoor conditions in large cages. These egg masses were obtained after lab rearing of insects collected in the field close to Versailles (48°48′19″N, 2°08′06″E, France) in 2013. Development of the larvae was followed for about 1 month until the larval stage L4 was reached, based on the size of the head capsule. Larvae were collected and frozen at − 80 °C before sRNA extraction. The samples comprised four conditions: On fed on corn, On fed on mugwort, Os on corn, Os on mugwort. Each experiment comprised two replicates. For *Spodoptera*, the reciprocal transplant experiment has been performed in similar but controlled conditions in a quarantine lab [13, 70].

Corn (Corn line B73) and rice (Arelate variety from CFR, Centre Français du Riz) were produced from organic seed at the DIASCOPE experimental research station (INRA, Mauguio, France, 43°36′37″N, 3°58′35″E) in plastic pots (7 × 8 cm for both plants in RT filled with conventional substrate). Mugwort rhizomes were sampled near Versailles in March 2013 and transplanted to 12.5-L plastic pots at the DIASCOPE station for further growth and experiments.

The terms maize/corn are used interchangeably throughout the manuscript.

The RT experiments with *Ostrinia* and *Spodoptera* have been performed on the same corn lines, by the same experimentator, RNA extraction has been performed on the same insect instar L4, the sequencing has been done by the same company MGX, the RNA-Seq analysis by the same method DESeq2. However since *S. frugiperda* is a quarantine organism in France, the RT experiment has been done inside the lab while in semi natural conditions for *Ostrinia*.

### Small RNA extraction and sequencing

sRNA extraction was performed on a pool of 15 L4 On or Os larvae per condition according to the protocol described in [17]. For construction of the libraries, sRNA were size-selected in the range of 15 to 40 nucleotides and sequencing was performed on Illumina HiSeq 2500 by the MGX -Montpellier GenomiX (Montpellier, France) generating sRNA reads of 50 nucleotides in length.

### miRNA genes annotation

Precursor sequences of miRNA were predicted using miRDeep2, an algorithm based on a probabilistic model which scores the fit of sequenced RNA to the biological model of miRNA biogenesis [71]. Raw reads were trimmed to remove adapter sequences using cutadapt software (version 1.4.1) [72] and aligned on the *Ostrinia scapulalis* genome assembly OSCA v1.2 [33] using the mapping module of the miRDeep2 software [73]. Only reads mapping less than 5 times were used further. Using read mappings as guidelines, putative miRNA precursors were excised and the miRDeep2 core algorithm scored their likelihood as real miRNA precursors. miRDeep2 [73] maps the sRNA reads (pools of either the On or the Os sncRNA reads in this case) to the genome and excises potential miRNA precursors sequences from the genome. Predictions of the secondary structures of the miRNA precursors and estimation of their stability were made using RNAfold. A probabilistic model of miRNA biogenesis by the Dicer protein is used by MirDeep2 to score frequency and compatibility of mapping of the sRNA sequence reads (representing “the signature”) on the secondary structure of the miRNA genomic precursors (which represents “the structure”) as compared to a non-miRNA precursor hairpin. Read stacks aligned on the structure correspond to mature miRNA sequences. The score represents the likelihood of each precursor to represent a genuine miRNA. However, the algorithm may generate false positives i.e. hairpins with read stacks unrelated to miRNA biology*.* To estimate the rate of false positives, the algorithm shuffles the observed combinations of structures and signatures and compares the score distribution between the genuine combinations and the control ones for varying score cut-offs [17, 71, 73]. The sequences of mature predicted miRNAs are compared to mature miRNA sequences contained in miRBase (release 21) which allows to sort them in two classes, known or novel depending if they are included or not in miRBase. The output is a scored list of known and novel miRNA in the deep sequenced sample. Known miRNAs were identified by similarity to miRNA sequences from miRBase database (release 21).

### Mapping of sncRNA reads on different reference sequences

Mapping was performed using Bowtie 1 [74], allowing one mismatch when reads of On were mapped on Os reference sequences. Counts of reads mapping at least once were used to make the homology diagram on Fig. 2e, f.

### Analysis of differential miRNA and target mRNA expression

The design of the RNA-Seq experiments (number of replicates, read depth) fits with criteria defined in the study of Lamarre et al., 2018 [37].

#### For miRNA

The miRDeep2 software provides the number of reads mapping to the predicted precursor miRNA. We used these counting data as input for the R package DESeq2 [35] to assess the variation in miRNA expression following a change of host-plant in On and Os or between On and Os species reared on the same plant, corn or mugwort. DESeq2 uses negative binomial generalized linear models to test for differential expression. An adjusted *p*-value for multiple testing was computed with the Benjamini-Hochberg procedure to control false discovery rate (FDR). Results with an FDR < 0.05 were considered statistically significant.

#### For mRNA

For that purpose, we re-analyzed RNA-Seq raw data obtained during the same RT experiment and published previously [13] with the DESeq2 software instead of EdgeR initially to allow comparison of miRNA targets between *Ostrinia* and *Spodoptera frugiperda.*

### Experimental validation of differential expression

The miRNA expression levels were quantified using TaqMan small RNA assay system from LifeTechnologies. Briefly, total RNA from samples was isolated using Trizol. After reverse transcription, the cDNA was used for qRT analysis with TaqMan probes according to the manufacturer’s instructions on two biological replicates of the RT experiment, with three technical replicates. The detailed protocol is available in reference [17]. The qRT-PCR analysis was performed using the 2-ΔΔCT method and each Ct value of the tested miR was normalized to that of an endogenous miRNA (mir-124) whose expression remained stable in the different experimental conditions based on read counts and DESeq2 analysis. We used a pairwise fixed reallocation randomization statistical test (2000 iterations, *p*-value< 0.001) [75] to check if gene expression varied significantly between two experimental conditions.

### Detection of potential target genes regulated by miRNA

To detect putative gene targets, we applied two different software, TargetScan [76, 77] and miRanda [78] on the 3’UTR of mRNA contigs discovered with exUTR pipeline [79] applied to On and Os contigs [NCBI BioProject accession number PRJNA392376]. We kept as candidates the genes that were predicted by the two software and which were differentially expressed in the same conditions (assessed by DESeq2; FDR < 0.05). TargetScan predicts biological targets of miRNAs by searching for the presence of conserved 8-mer and 7-mer sites that match seed region of each miRNA by calculating thermodynamic free energy using the RNAFold package [77]. Predictions are ranked using the site number, site type, and site context. TargetScan (version 5.0) was run with default parameters. miRanda (version v3.3a) [78] allows one wobble pairing in the seed region when it is compensated by matches in the 3′ end of the miRNA, it calculates the binding energy of the duplex structure and its position within the 3’UTR, it was used with the same parameters as in [17].

## Supplementary Information

**Additional file 1: Supplementary Table S1.** Number of sequence reads in each small non coding RNAs library.

**Additional file 2: Supplementary Excel Table S2.** Predictions of miR genes by mirDEEP2 in On and Os and orthology table.

**Additional file 3: Supplementary Figure 1.** Base composition of known or novel mature miRNAs using weblogo [79, 80].

**Additional file 4: Supplementary Figure 2.** MA-plots showing the relative expression of known or novel miR according to the host-plant (Mugwort compared to corn) in each sibling species. Top panel, in On, bottom panel In Os.

**Additional file 5: Supplementary Figure 3.** Variation between samples (treatments, replicates) of larvae exposed to different plants displayed by Principal Component Analysis (PCA). Top panel: On, bottom panel: Os. OnCor: On on corn, OnMug: On on mugwort, OsCor: Os on corn, OsMug: Os on mugwort.

**Additional file 6: Supplementary Excel Table S3.** Relative expression resulting from DESEQ2 analysis within sibling species according to the host-plant.

**Additional file 7: Supplementary Figure 4.** MA-plots showing the relative expression of known or novel miR according to the genetic background. Relative expression analyzed by DESEQ2 in Os compared to On on corn.

**Additional file 8: Supplementary Excel Table S4.** Relative expression resulting from DESEQ2 analysis according to the genetic background on the same host-plant.

**Additional file 9: Supplementary Figure 5.** Variation between samples (treatments, replicates) of larvae exposed to different plants displayed by Principal Component Analysis (PCA). Os compared to On on corn. OnCor: On on corn, OnMug: On on mugwort, OsCor: Os on corn, OsMug: Os on mugwort.

**Additional file 10: Supplementary Excel Table S5.** List of gene target predictions of known miRs of On and Os predicted both by MiRanda and TargetScan on mRNA contigs with 3’UTRs.

**Additional file 11: Supplementary Excel Table S6.** List of DE target genes (In On fed on mugwort compared to corn, FDR < 0.05) of known miRs. Their relative expression (column D to I) and their gene annotation (right part of the Table from AF to AH) is shown. Since many genes are targeted by multiple miRNAs, we provide also the list of unique genes (tag “without duplicates”), and the subset of gene targeted by DE miRs.

**Additional file 12: Supplementary Excel Table S7.** List of DE target genes (in Os compared to On on corn, FDR < 0.05) of known miRs. Their gene annotation (right part of the Table) is shown. Since many genes are targeted by multiple miRNAs, we provide also the list of unique genes inferred from gene IDs (tag “without duplicates”).

**Additional file 13: Supplementary Excel Table S8.** List of DE target genes (FDR < 0.05) of known miRs predicted by TargetScan and by MiRanda. Their relative expression in Sf-C on plants (Tag “within Sf-C”, column W to AB) or in Sf-R compared to Sf-C on corn (Tag “between strains on corn” (column Y to AD)) and their gene annotation (right part of the Table) is shown. Since many genes are targeted by multiple miRNAs, we provide also the list of unique genes that are targeted by miRs (Tag “without duplicates”).

**Additional file 14: Supplementary Excel Table S9.** Summary of DE miR gene targets shared between Ostrinia and Spodoptera.

**Additional file 15: Supplementary Excel Table S10.** Comparison of known miRs predicted in On and Sf-C.

## Data Availability

Dataset for *S. frugiperda* RNAseq [1] is available in Array Express: E-MTAB-6540. Dataset for *Ostrinia* miRNAs is available in Array Express: E-MTAB-10014. http://www.ebi.ac.uk/arrayexpress/help/how_to_search_private_data.html For reviewer access from 2021 to 01-14 at about 6 am UK time, please use the following login details. Username: Reviewer_E-MTAB-10014. Password: 66VvhpXh.
