# Supplementary material for: Integrated miRNA and transcriptome profiling to explore the molecular determinism of convergent adaptation to corn in two lepidopteran pests of agriculture
Source: BMC Genomics. 2021 Aug 9;22:606. doi: 10.1186/s12864-021-07905-7 (PMC8351448; doi:10.1186/s12864-021-07905-7)
Supplement: Supplementary file 1 — Additional file 1: Supplementary Table S1. Number of sequence reads in each small non coding RNAs library. [file 12864_2021_7905_MOESM1_ESM.docx]

S**upplementary Information of the manuscript entitled:**

Integrated miRNA and transcriptome profiling to explore the molecular determinism of convergent adaptation to corn in two lepidopteran pests of agriculture.

**Authors**

Sylvie Gimenez*, Imène Seninet*, Marion Orsucci, Philippe Audiot, Nicolas Nègre, Kiwoong Nam, Réjane Streiff, and Emmanuelle d’Alençon.

* Co-first authors, these authors contributed equally to the work

| Samples* | CB* | CAF* | % (/CB) | Unique reads |
| --- | --- | --- | --- | --- |
| OnCor 1  (tube3) | 27 237 923 | 24 132 247 | 88.60 | 2438275 |
| OnCor 2  (tube 4) | 22 824 927 | 20 297 128 | 88.93 | 2399427 |
| OnMug 1  (tube 1 ) | 59 228 699 | 52 191 005 | 88.01 | 1252299 |
| OnMug 2  (tube 2 ) | 61 354 896 | 54 001 034 | 88.12 | 1596536 |
| OsCor 1  (tube 7) | 60 379 738 | 53 107 111 | 87.96 | 1335321 |
| OsCor 2  (tube 8) | 28 307 217 | 25 258 716 | 89.23 | 1224794 |
| OsMug1  (tube5) | 17 321 698 | 15 349 259 | 88.61 | 1377834 |
| OsMug 2  (tube 6) | 11 434 208 | 10 383 562 | 90.81 | 732631 |

* OnCor: On on corn, OnMug: On on mugwort, OsCor: Os on corn, OsMug: Os on mugwort.

*CB : Number of raw clusters

*CAF : Number of clusters after filtering and adapter trimming
