# Supplementary figures and images for: Integrated miRNA and transcriptome profiling to explore the molecular determinism of convergent adaptation to corn in two lepidopteran pests of agriculture
Source: BMC Genomics. 2021 Aug 9;22:606. doi: 10.1186/s12864-021-07905-7 (PMC8351448; doi:10.1186/s12864-021-07905-7)

## Slide 1
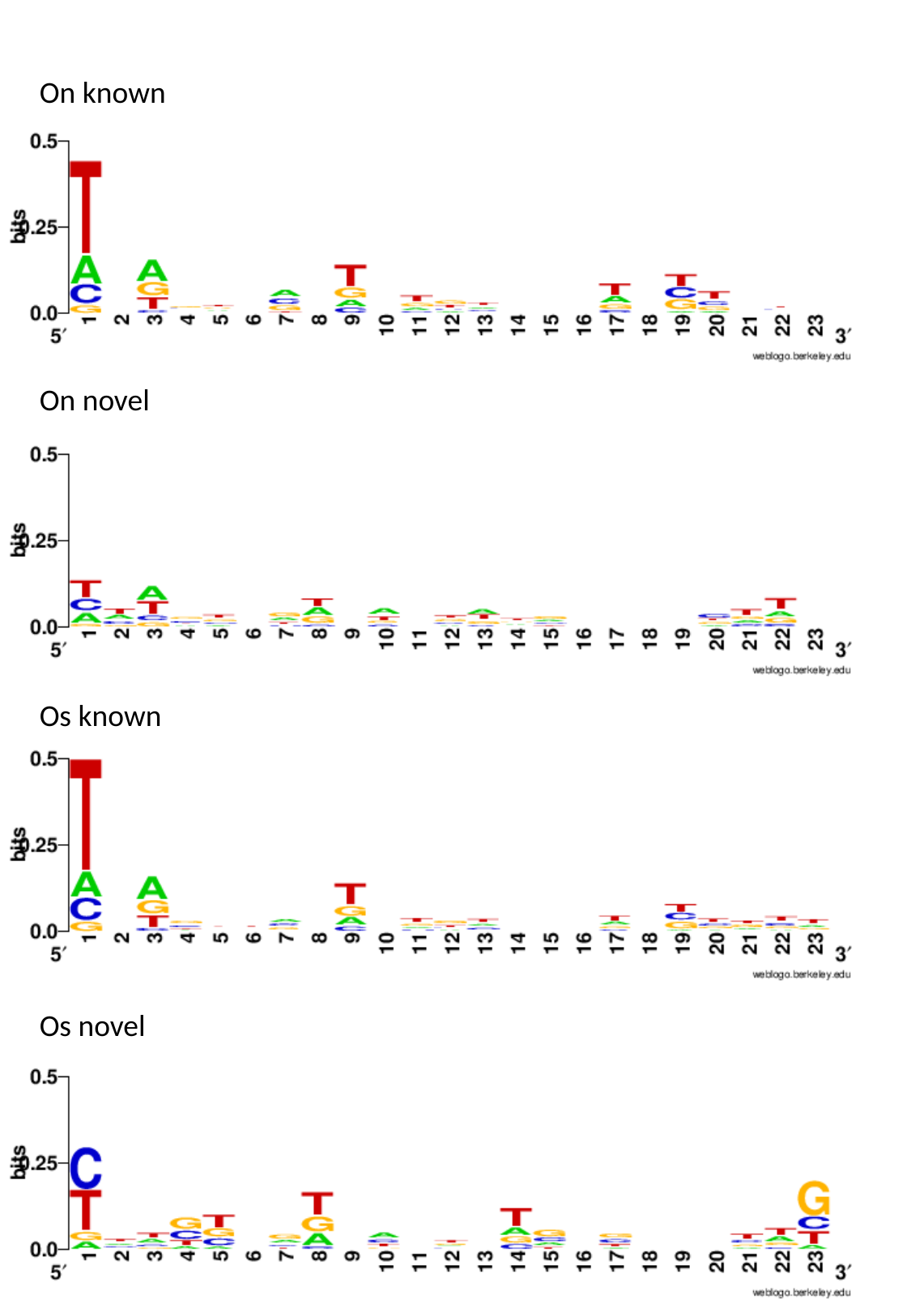

On known
On novel
Os known
Os novel

Supplement: Supplementary file 3 — Additional file 3: Supplementary Figure 1. Base composition of known or novel mature miRNAs using weblogo [79, 80]. [file 12864_2021_7905_MOESM3_ESM.pptx]

## Slide 1
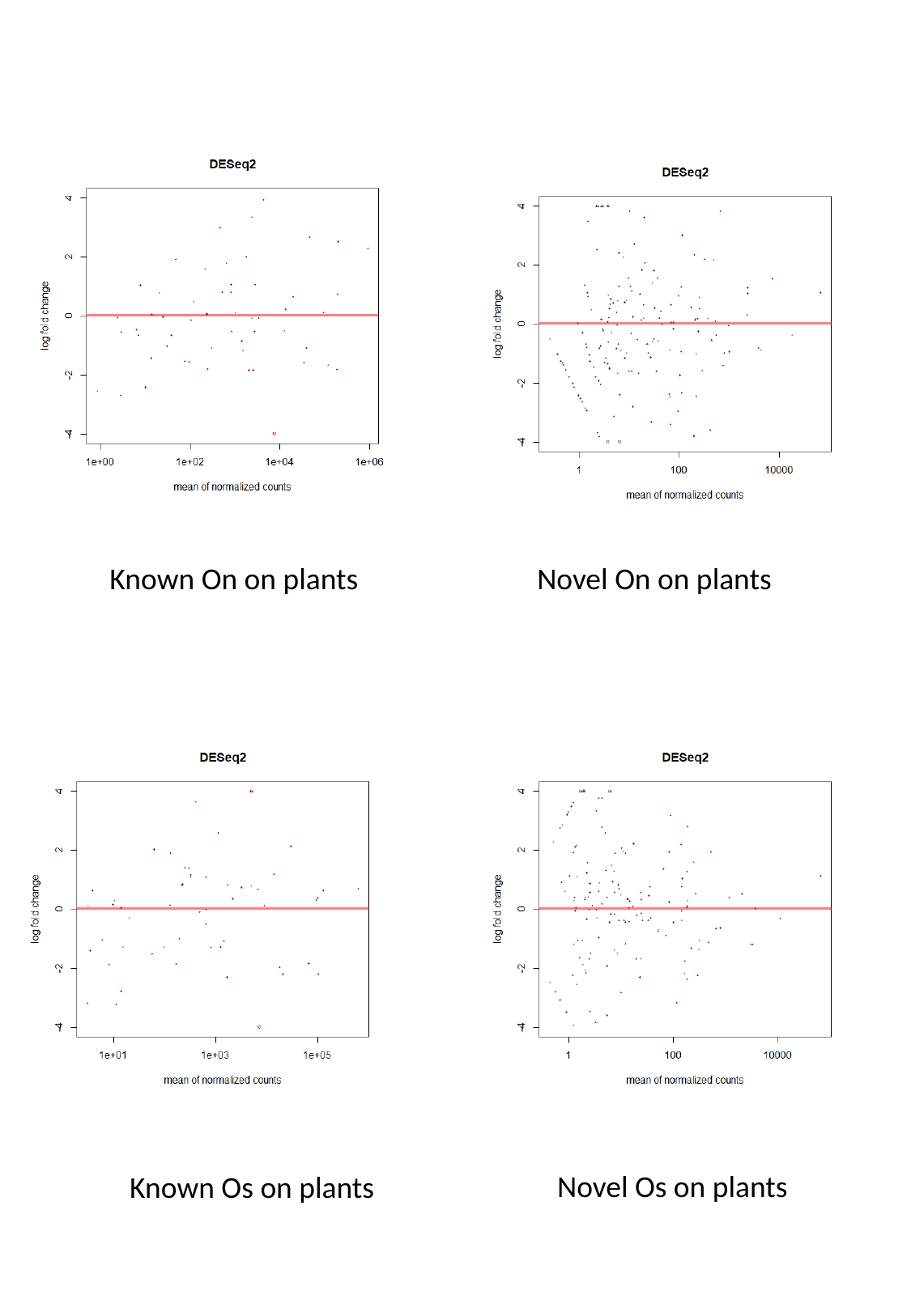

Novel On on plants
Known On on plants
Novel Os on plants
Known Os on plants

Supplement: Supplementary file 4 — Additional file 4: Supplementary Figure 2. MA-plots showing the relative expression of known or novel miR according to the host-plant (Mugwort compared to corn) in each sibling species. Top panel, in On, bottom panel In Os. [file 12864_2021_7905_MOESM4_ESM.pptx]

## Slide 1
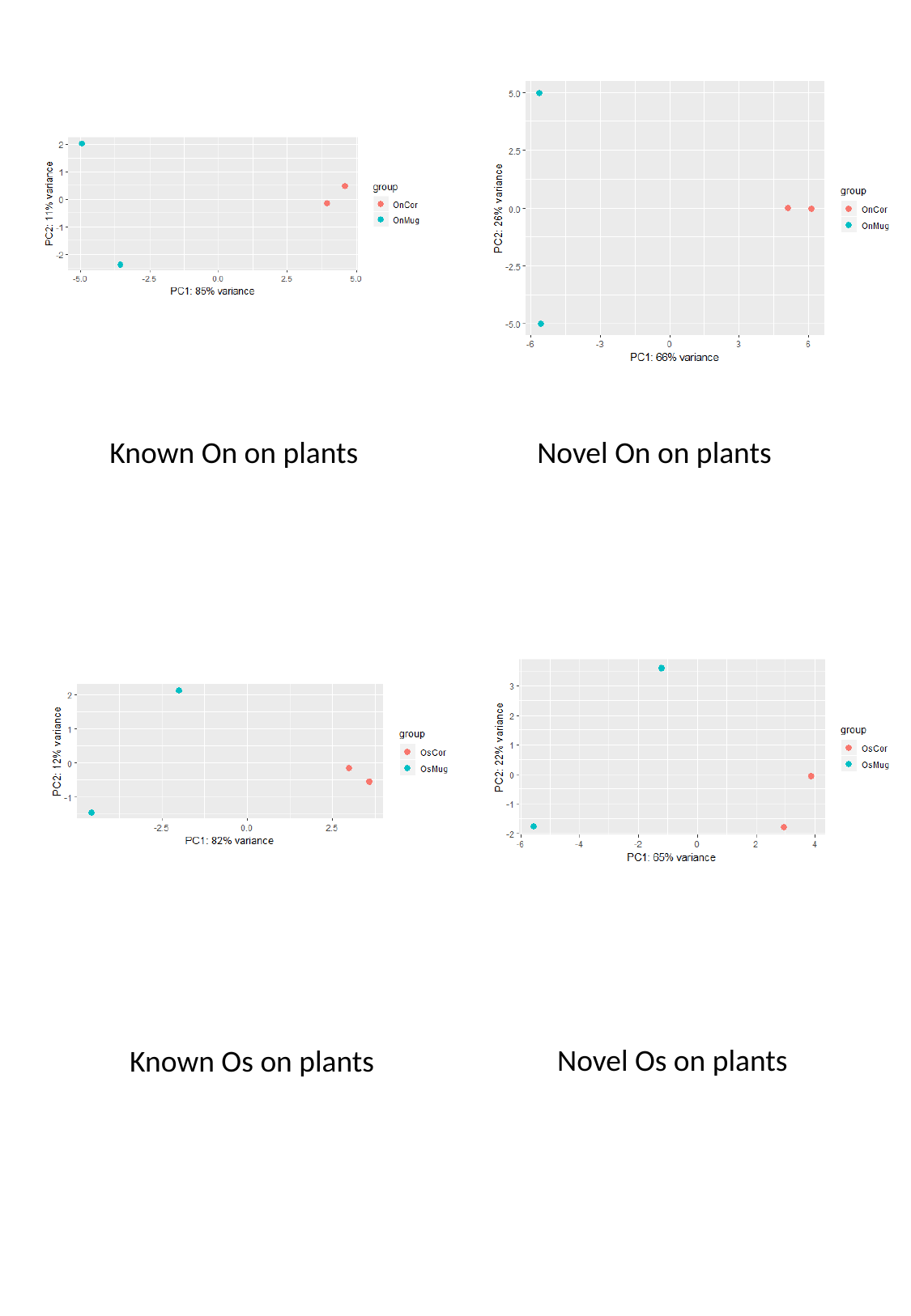

Novel On on plants
Known On on plants
Novel Os on plants
Known Os on plants

Supplement: Supplementary file 5 — Additional file 5: Supplementary Figure 3. Variation between samples (treatments, replicates) of larvae exposed to different plants displayed by Principal Component Analysis (PCA). Top panel: On, bottom panel: Os. OnCor: On on corn, OnMug: On on mugwort, OsCor: Os on corn, OsMug: Os on mugwort. [file 12864_2021_7905_MOESM5_ESM.pptx]

## Slide 1
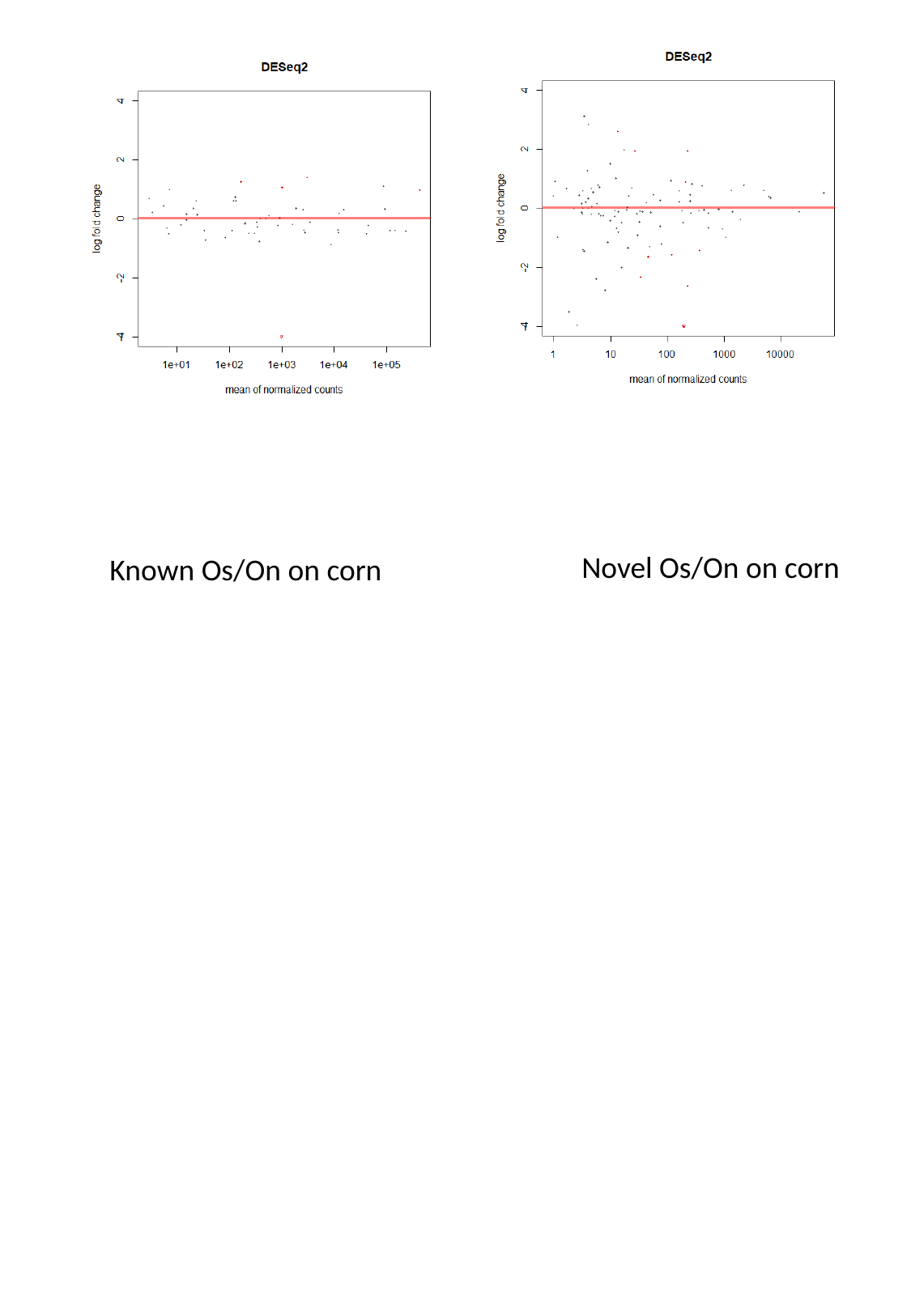

Novel Os/On on corn
Known Os/On on corn

Supplement: Supplementary file 7 — Additional file 7: Supplementary Figure 4. MA-plots showing the relative expression of known or novel miR according to the genetic background. Relative expression analyzed by DESEQ2 in Os compared to On on corn. [file 12864_2021_7905_MOESM7_ESM.pptx]

## Slide 1
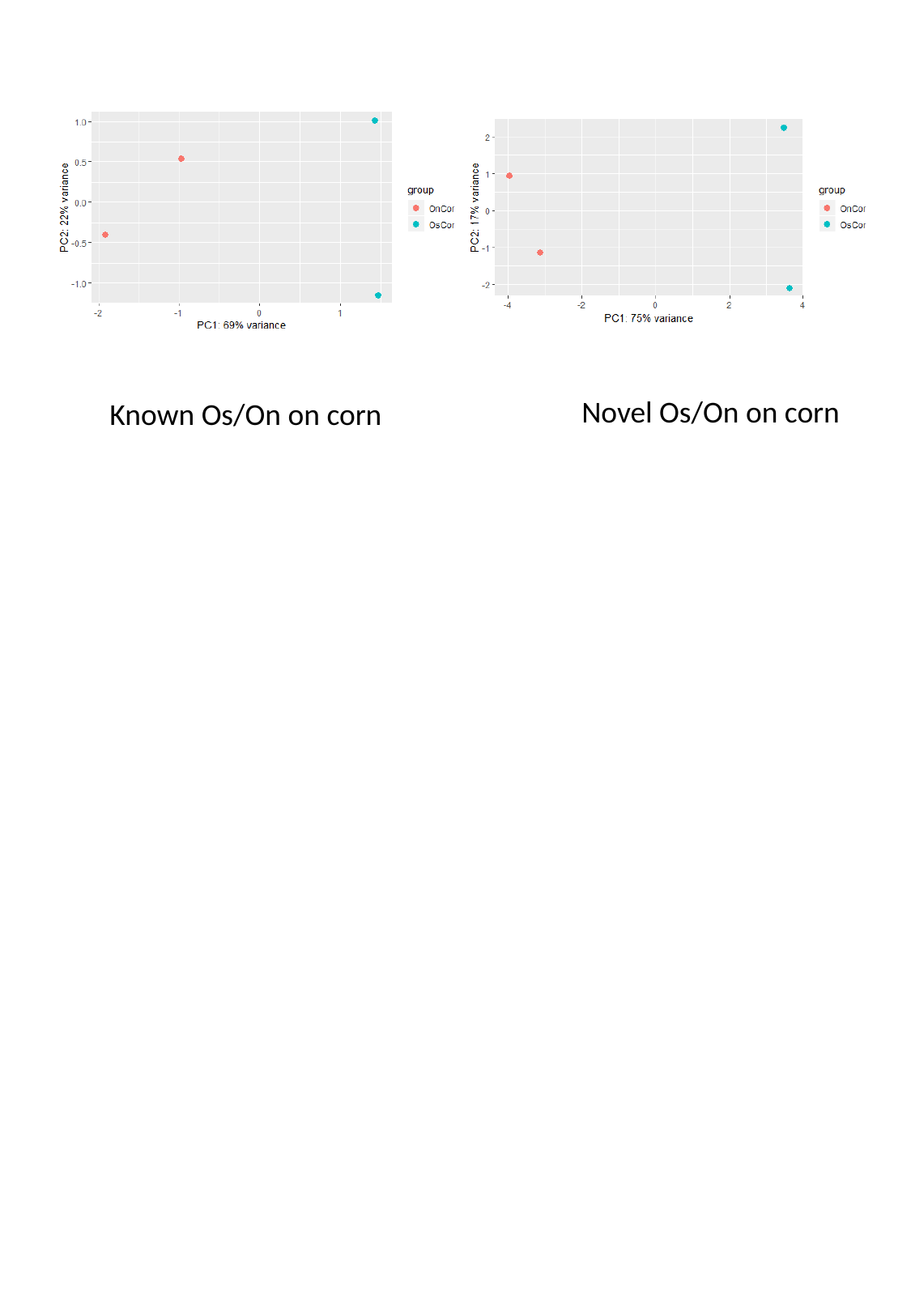

Novel Os/On on corn
Known Os/On on corn

Supplement: Supplementary file 9 — Additional file 9: Supplementary Figure 5. Variation between samples (treatments, replicates) of larvae exposed to different plants displayed by Principal Component Analysis (PCA). Os compared to On on corn. OnCor: On on corn, OnMug: On on mugwort, OsCor: Os on corn, OsMug: Os on mugwort. [file 12864_2021_7905_MOESM9_ESM.pptx]
